# Galantamine Based Novel Acetylcholinesterase Enzyme Inhibitors: A Molecular Modeling Design Approach

**DOI:** 10.3390/molecules28031035

**Published:** 2023-01-19

**Authors:** Luciane B. Silva, Elenilze F. B. Ferreira, Maryam, José M. Espejo-Román, Glauber V. Costa, Josiane V. Cruz, Njogu M. Kimani, Josivan S. Costa, José A. H. M. Bittencourt, Jorddy N. Cruz, Joaquín M. Campos, Cleydson B. R. Santos

**Affiliations:** 1 Graduate Program in Medicinal Chemistry and Molecular Modeling, Health Science Institute, Federal University of Pará, Belém 66075-110, Brazil; 2 Laboratory of Modeling and Computational Chemistry, Department of Biological and Health Sciences, Federal University of Amapá, Macapá 68902-280, Brazil; 3 Laboratory of Organic Chemistry and Biochemistry, University of the State of Amapá, Macapá 68900-070, Brazil; 4 COMSATS University Islamabad, Park Road, Tarlai Kalan, Islamabad 45550, Pakistan; 5 Department of Pharmaceutical Organic Chemistry, Faculty of Pharmacy, Campus of Cartuja, University of Granada, 18071 Granada, Spain; 6 Laboratory of Biotechnology in Natural Products, Department of Biological and Health Sciences, Federal University of Amapá, Macapá 68902-280, Brazil; 7 Department of Physical Sciences, University of Embu, Embu P.O. Box 6-60100, Kenya

**Keywords:** Alzheimer’s disease, ADME, molecular docking and molecular dynamics

## Abstract

Acetylcholinesterase (AChE) enzymes play an essential role in the development of Alzheimer’s disease (AD). Its excessive activity causes several neuronal problems, particularly psychopathies and neuronal cell death. A bioactive pose on the *h*AChE B site of the human acetylcholinesterase (*h*AChE) enzyme employed in this investigation, which was obtained from the Protein Data Bank (PDB ID 4EY6), allowed for the prediction of the binding affinity and free binding energy between the protein and the ligand. Virtual screening was performed to obtain structures similar to Galantamine (GNT) with potential *h*AChE activity. The top 200 hit compounds were prioritized through the use of filters in ZincPharmer, with special features related to the pharmacophore. Critical analyses were carried out, such as hierarchical clustering analysis (HCA), ADME/Tox predictions, molecular docking, molecular simulation studies, synthetic accessibility (SA), lipophilicity, water solubility, and hot spots to confirm the stable binding of the two promising molecules (ZINC16951574-LMQC2, and ZINC08342556-LMQC5). The metabolism prediction, with metabolites M3-2, which is formed by Glutathionation reaction (Phase II), M1-2, and M2-2 formed from the reaction of S-oxidation and Aliphatic hydroxylation (Phase I), were both reactive but with no side effects. Theoretical synthetic routes and prediction of synthetic accessibility for the most promising compounds are also proposed. In conclusion, this study shows that in silico modeling can be used to create new drug candidate inhibitors for *h*AChE. The compounds ZINC16951574-LMQC2, and ZINC08342556-LMQC5 are particularly promising for oral administration because they have a favorable drug-likeness profile, excellent lipid solubility, high bioavailability, and adequate pharmacokinetics.

## 1. Introduction

Alzheimer’s disease (AD), discovered and named by the German scientist Alois Alzheimer (1907), is a progressive neurodegenerative disease. In the world, approximately 47 million people are affected by this disease, which is projected to increase by 62% before 2050 [1]. As a neurodegenerative, progressive, irreversible, AD develops due to the loss of neurons in the central nervous system and the malfunctioning of nerve cells. Early-onset AD (EOAD) is generally hereditary autosomal dominant, constituting only 1–2% of AD, with genes including amyloid precursor protein (APP), presenilin 1 (PSEN1), and presenilin 2 (PSEN2) being considered the main factors. There is precedence in the literature for identifying two different types of mutations in this gene in patients with familial AD [2]. Several risk factors have been associated with Alzheimer’s disease, such as neuritic plaques or amyloid plaques and trisomy 21, a risk factor for early-onset dementia, in addition to rapid loss of synapses and degeneration of baseline cholinergic neurons [3].

Clinically, the most common diagnosis is indicated by progressive cognitive deficiencies or defined by loss of memory and learning ability, making it impossible to perform routine activities, and an adverse set of neuropsychiatric symptoms, such as apathy, verbal and physical agitation, irritability, anxiety, depression, delusions and hallucinations [4].

Besides, among the mechanisms related to the appearance and evolution of AD, the “cholinergic hypothesis” has emerged as a widely accepted therapeutic means to improve cognitive functions in AD, and different studies suggest that cholinergic inputs are present in this process [5]. It has been observed that cholinergic activity can influence amyloid processing. In the absence of muscarinic receptor activity, the amyloidogenic pathway is privileged, while the opposite occurs with regular receptor activity [6]. Another hypothesis is the “amyloid cascade,” which was first proposed in 1992. Other conjectures, such as the oligomeric and metallic hypotheses, can be extensions of the amyloid hypothesis and began taking on more significant proportions in the 1990s [7].

In recent years, with a comprehensive discussion about the amyloid cascade hypothesis, a growing body of evidence has come to suggest that endogenous metal ions, particularly those that have redox activity, such as copper (II) and iron (III), in addition to certain non-redox-active ions, such as zinc (II), can contribute to the evolution of neurodegenerative diseases, favoring the aggregation of Aβ and increasing its toxicity [8,9].

AD is also associated with the deficit of the neurotransmitter Acetylcholine (ACh) and oxidative stress caused by an exacerbation of glutamatergic transmission [10].

Acetylcholinesterase (AChE) is the essential enzyme in the serine hydrolases family in cholinergic synapses that plays a crucial role in memory and cognition [11]. AChE inhibition has also been recognized as a therapeutic strategy for other disorders, such as dementia, myasthenia gravis, glaucoma, and Parkinson’s disease, in addition to AD [12].

**Figure 1:**
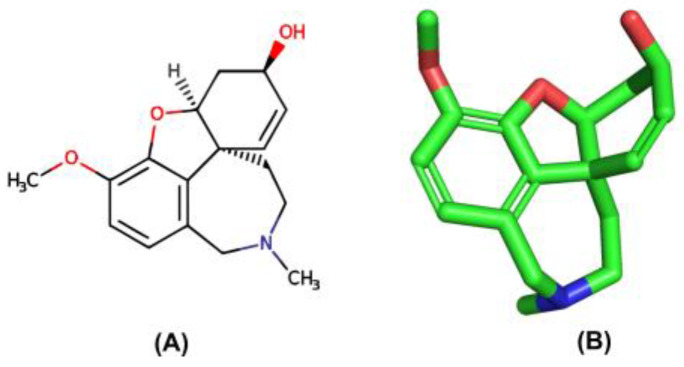
(**A**) 2D and (**B**) 3D structure of the GNT compound. Green are carbon atoms, red oxygen atoms and blue nitrogen atoms.

Currently, AChE inhibitors, including Donepezil (Aricept^®^), Galantamine (GNT) (Reminyl^®^) (Figure 1), and Rivastigmine (Exelon^®^), are widely used in symptomatic treatments for AD. However, the effectiveness of these drugs is hindered by their side effects, such as gastrointestinal disorders, hepatotoxicity, dizziness, diarrhea, vomiting, nausea, pharmacokinetic disadvantages, and hypotension [4]. Thus, it is necessary to discover new, more effective compounds to reduce the risk.

**Figure 2:**
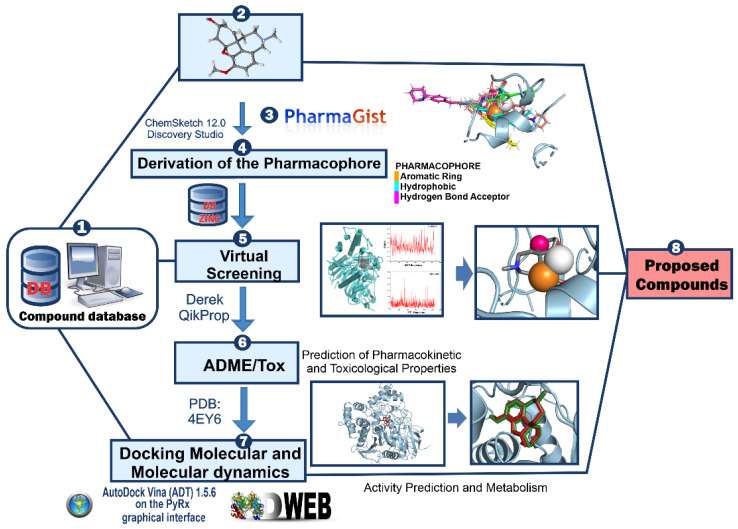
Main methodological steps. (1) Database to search for the compounds, (2) (3) and (4) generation and validation of the pharmacophoric model, (5) virtual screening, (6) evaluation of the ADME/Tox in silico properties of the selected compounds, (7) evaluation of the mode of interaction by docking and molecular dynamics and finally (8) proposed compounds.

Thus, this paper uses molecular modeling approaches to select new compounds with potential *h*AChE inhibition capacity. For this, we use the GNT as a control to filter new compounds. We sought to obtain new molecules with potential inhibitory activity hAChE via a pharmacophore model generation. The generated pharmacophore model was used to screen chemical databases virtually. The successful compounds were filtered by evaluating their drug-like properties, which were statistically assessed using Pearson’s correlations and hierarchical clustering analysis (HCA). Subsequently, they were submitted to pharmacokinetic, toxicological, and biological activity predictions. The successful compounds’ binding mode analyses were performed using docking and molecular dynamics simulations. Once identified, the compounds were subsequently subjected to metabolism prediction, ADME/Tox to metabolites, hot spots, and theoretical synthetic routes proposed for the most promising compounds. The methodological scheme of the steps used in this study is shown in Figure 2.

## 2. Results and Discussion

### 2.1. Pharmacophore Model Generation

In PharmaGist the 15 selected molecules (Figure S1 in Supplementary Materials) were used as the input, and GNT was added as a template, with a score of 32.692. Subsequently, a matrix with the following pharmacophoric descriptors: atoms (ATM), spatial characteristics (SF), aromatic (ARO), hydrophobic (HYD), and Hydrogen-bond acceptor (ACC) was constructed, and their values evaluated using the statistical software STAT.

The statistical method of Pearson’s correlation aimed to show the correlation between pharmacophoric descriptors and the inhibitory activity of the molecules (*p*IC_50_ = −logIC_50_) so that it was possible to evaluate between the correlations which values should be considered in the analysis as described by Ferreira et al. 2019 [13].

The pharmacophore descriptors and the biological activity described have a proportional correlation. For instance, a Hydrogen-bond acceptor (ACC) with a *p*-value of −0.804 shows the number of hydrogen acceptors that correlate with the *p*IC_50_ response of the selected molecules. Significantly, as with the descriptor Atoms (ATM) with a value of *p* = −0.634, each pharmacophoric descriptor contributes to an inhibitory activity (*p*IC_50_).

Also, a note from Table 1, the correlation between pharmacological drug parameters is less than 0.928. In contrast, the correlation between inhibitory activity (*p*IC_50_) is more significant than −0.607, that is, an inversely proportional correlation, especially to the Hydrogen-bond acceptor (ACC). The selected pharmacophoric characteristics represent the necessary characteristics for generating pharmacophoric models in the search to identify potential compounds with AChE enzyme inhibitory activity.

### 2.2. Evaluation of the Pharmacophore Model

**Figure 3:**
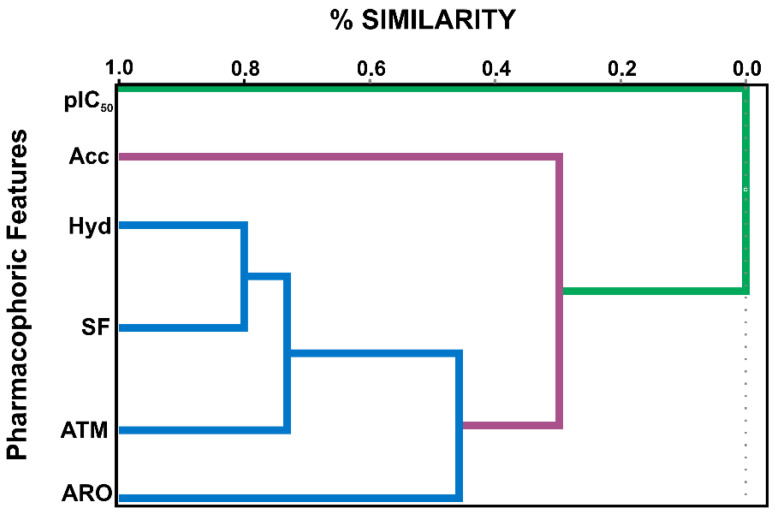
HCA dendrogram, a correlation between pharmacophoric characteristics and *p*IC_50_.

From the result of the Pearson correlation, the model can be evaluated by a chemometric study. A dendrogram (Figure 3) was obtained from a hierarchical clustering analysis (HCA) using the Pirouette 4.0 software. Confirmation of the data obtained by Pearson’s correlation was conducted by the generation of HCA pharmacophoric hypotheses in which a correlation of the biological activity (*p*IC_50_) as an independent variable and the structural similarity cursor in categories, namely, more active (a) and less active (b), originating from six molecular descriptors, including Atoms (ATM), Spatial characteristics (SF), aromatic (ARO), hydrophobic (HYD), and Hydrogen-bond acceptor (ACC), was performed (Figure 3).

The descriptor Hydrogen-bond acceptor (ACC) in the pink cluster shows a greater approximation with the *p*IC_50_. This confirms the value obtained in the Pearson correlation. The descriptors Hydrophobic (HYD), Spatial Resources (SF), Atom (ATM), and aromatic (ARO) are grouped into the same cluster (in blue).

**Figure 4:**
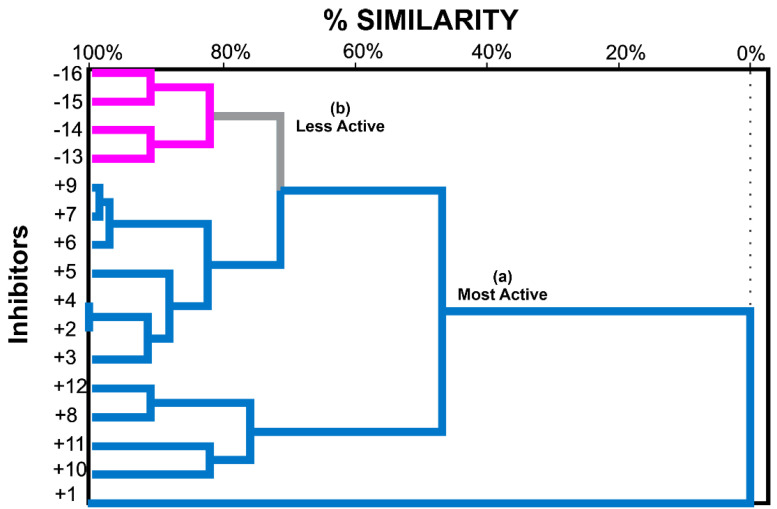
HCA dendrogram for 15 molecules derived from Gal with potential inhibitory activity of *h*AChE and GNT identified as number 1, the clusters are divided into (a) Most active represented in blue, and (b) less active, represented in pink.

The HCA technique showed a similar dendrogram in which the molecules were classified into two classes (more active and less active) according to their similarities. In the largest cluster, called the most active (in blue), are molecules 1–12 with the best inhibitory activity values. In the smallest cluster (in pink), molecules 13–16 are the least active (Figure 4).

Thus, by the dendrogram in Figure 4, it is quickly perceived that molecules 1 and 9 are the most distinct compared to the other most active molecules (compounds 1, 2, and 3). However, they are very close, indicating a high chemical similarity.

In addition, the Hydrogen-bond acceptor (ACC), Hydrophobic (HYD), and Atoms (ATM) acceptance number is a better prediction observed by molecule 10 (5 ACC, 17 HYD, and 109 ATM), which shows the highest values, as well as 11, 8, 12. Molecules +2 and +4 that are highly similar and have the same ACC, HYD, and ATM values also have better predictions. It is noticed that for the most active molecules, the descriptors ATM and HYDRO resources are relevant to the value of the inhibitory activity, shifting them to the most active side, confirming the importance of the number of Hydrogen-bond acceptors (ACC). However, in molecules +7 and +9 having the same number of ACC and HYD, their inhibitory activity is accentuated by the number of atoms in their structure (80 and 82).

It is possible to observe the presence of the indole group in the most active molecules by the similarity that ends the side chain linked to the N atom. The indole fraction of one of the molecules binds in the same region, where Aᵦ join *h*AChE, where they share the same binding site as GNT [14]. The indole fraction was selected as a suitable parameter for binding to aromatic residues in PAS due to its ability to participate in hydrophobic interactions [15].

### 2.3. Pharmacophoric-Based Hierarchical Virtual Screening

**Figure 5:**
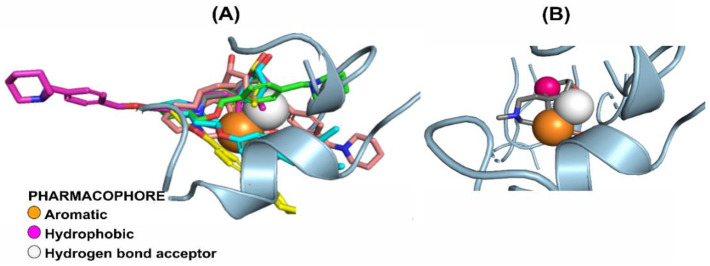
Drug model selected for virtual screening. Orange sphere (Aromatic Ring), Pink (Hydrophobic), and White (Hydrogen-bond acceptor). (**A**) The pharmacophoric hypothesis was tested on a set of selected compounds. (**B**) Hypothesis and pharmacophoric analysis of the active site: the three characteristics are in regions of interactions with seven essential amino acids at the *h*AChE receptor.

The pharmacological model obtained through PharmaGist was submitted to the ZINCpharmer server to obtain the spatial coordinates of the pharmacophore. The aligned molecules shared 3 pharmacophoric characteristics: Aromatic (ARO), Hydrophobic (HYD), and Hydrogen-bond acceptor (ACC) (Table 2 and Figure 5), generating a model with the following coordinates:

As a result of the virtual screening used in medicines, Top Hit 200 compounds were prioritized through filters in ZincPharmer, with special features related to the pharmacophore. Success for the compound shows that hAChE inhibitory activity depends on the precision and specificity of the activated pharmacophore [13]. ZINC includes 1.4 billion compounds, which continue to grow in size with ZINC20 and 1.3 billion of which are purchasable, sourced from 310 catalogs from 150 companies. Over 90% of catalogs are refreshed every 90 days, and over 90% of compounds have been verified as purchasable within the last three months [16]. A virtual screening through the library composed of commercial ZINC molecules and internal molecules (real/virtual files extended from the real scaffold) as an internal 3D database prepared for the virtual tracking of any model [16]. Thus, a molecular adjustment was applied to filtered molecules from the virtual screening based on the pharmacophore model.

### 2.4. Pharmacokinetic Predictions

Our pharmacokinetic selection was designed to determine a molecule with physicochemical properties equal to or better than the reference molecule, GNT. In Table 3, it can be seen that the predictions of the pharmacokinetic properties of the selected molecules from the virtual screening reduced the number of compounds in a top hit 200 to obtain, in this phase, 8 molecules through the following physical-chemical descriptors.

The #Star parameter evaluated the pharmacokinetic profile of the compounds with a general pharmacokinetic acceptance rating for the drug similarity parameter, which indicates property descriptors for value ranges optimized for 95% of the drugs [17]. In this analysis, all selected compounds showed values equal to zero, i.e., no violation, indicating an important similarity with the commercially available drugs. However, GNT exhibited 1 violation within these parameters (Star). There were no violations of Lipinski’s rule of five, according to the parameter for which all the molecules have values equal to zero [18].

Additionally, all the molecules have favorable properties concerning the percentage of oral absorption, a desired characteristic for developing drug candidates that can be efficient for oral administration. This data was corroborated by the cell models QPP_Caco_ (nm/s) and QPP_MDCK_ (nm/s), which also refers to the prediction of oral absorption of the drug, used as a model for the intestinal blood barrier. The QPP_Caco_ results show values above 1838.444, more than double that of the reference molecule (GNT), values that estimate excellent intestinal absorption. Since the permeability of the blood-brain barrier cells is stimulated by the MDCK cells (QPP_MDCK_), all compounds exhibited good results within parameters considered to be above 500 nm/s.

For the results obtained by the parameter QplogBB (blood and brain partition), values less than 1 must be used, and for QPlogPo/w (octanol/water), values must be less than 5; Positive values of the log BB mean that these compounds prefer brain tissue to blood, while a value close to zero means that a compound has a Brain/Blood concentration ratio, respectively. The results of QPlogBB for selected compounds showed values less than 0.611, while QPlogPo/w values were less than 3.828; both are good predictions for the compounds [17]. MW, corresponding to the molecular weight of the molecule in (g·mol^−1^), must obey the molar mass (MW) ≤ 500 g/mol for good oral bioavailability. The analysis of MW distributions showed a peak value between 208.257 and 350.234 g·mol^−1^. All the compounds do not violate this MW parameter, presenting values for good oral availability.

### 2.5. Toxicological Predictions

Derek software [19,20] classifies potentially toxic compounds into six categories: right, probable, plausible, implausible, improbable, or impossible (Table 4). The compounds were classified in the plausible category. A few selected compounds were similar to the reference molecule (GNT) and showed no toxicity alerts. The molecules that showed the best results in this step are ZINC86196920, ZINC16951574 (LMQC2), ZINC08342556 (LMQC5), ZINC86199797, ZINC13362890, and ZINC21657754. ZINC15910273 molecule generated a toxicophoric alert of skin sensitization due to the presence of hydrazine or precursor in its structures. A structural skin sensitization alert within a molecule indicates which molecule has the potential to cause skin sensitization. Whether a molecule is a skin sensitizer will also depend on its percutaneous absorption, having lipophilic characteristics that are absorbed more quickly by the skin and, therefore, are more likely to cause sensitization [21]. Therefore, these molecules were excluded due to their undesired potential toxic activities.

The ProTox-II [22] was used to determine the predicted oral toxicity of compounds based on 2D structure similarities with 33,000 compounds and their associated LD_50_ values, organ toxicity (hepatotoxicity), and toxicological endpoints (such as cytotoxicity) (Table 4). The predicted rodent LD_50_ was lowest for ZINC08342556 (LMQC5) at 1000 mg/kg with inactivity for hepatotoxicity and class IV, with most other candidates in the 362–800 mg/kg range. By comparison, the predicted LD_50_ of Pro-Tox-II for GNT was 85 mg/kg and class III (Table 4). However, only the molecules ZINC86196920, ZINC16951574 (LMQC2), ZINC08342556 (LMQC5), ZINC13362890, ZINC86199797, and ZINC21657754 presented class IV—if ingested, harmful (Table 4). Compounds ZINC13108311 and ZINC15910273 presented LD_50_ of 1000 mg/kg and class IV, but showed hepatotoxicity, corroborating the results obtained in the Derek of the toxicophoric alert. Additionally, the compound ZINC21657754 also showed active hepatotoxicity.

### 2.6. Prediction of Biological Activity (PASS)

The prediction of potential biological activity was performed using PASS [23] resulting in six selected compounds. Table 5 shows the biological activities that were considered: a CYP2D6 substrate, a CYP3A4 substrate, treatments for Alzheimer’s disease, and a CYP3A4 inducer. The values of Pa and Pi can vary from 0.000 to 1000, recorded as the compound’s probability of being inactive or active, respectively [24]. The biological activities obtained from the PASS were related to aspects of similarity with other bioactive substances.

Our results revealed that GNT has activity against the substrate CYP2D6 (Pa = 0.957) and CYP3A4 (Pa = 0.553) and treatment of Alzheimer’s disease (Pa = 0.428), confirming the results in silico. AChE has low pharmacokinetics in its interactions. However, since GNT is metabolized in the liver through CYP2D6 and CYP3A4, its hepatic metabolism can be affected by specific substrates, inhibitors, or enhancers of the same enzymes [25].

According to Table 5 and Table 6, the biological activity of these compounds revealed that ZINC86196920, ZINC16951574 (LMQC2), and ZINC86199797 have CYP2D6 and CYP3A4 substrate activities, all with Pa ˃ 0.5. This implies they have the greatest chance of being similar to other bioactive compounds. However, compounds with Pa < 0.5 did not show any reports about their activity in the database, that is, they do not have reported activities in the literature; they were also selected for the next stages of the process when their Pa ˃ Pi, as reported by Rodrigues et al. 2013 [26].

It is important to remember that the ZINC16951574 (LMQC2) proposal has a relatively good Pa value. At the same time, Pi tends to be 0 with high relevance and exhibits activities for the substrate CYP2D6 (Pa = 0.408) and CYP3A4 (Pa = 0.413). In addition to the results obtained from the in vitro study [27], this suggests that CYP2D6 and CYP3A4 metabolize about 75% of the drug. This proposal also contains the fact that it is modeled to respect the physicochemical, steric, and electronic properties important for the purpose for which these molecules were designed.

### 2.7. Evaluation of Generated Metabolites and Investigation of Their Properties ADMETox

#### 2.7.1. Metabolism Prediction of the Most Promising Compounds

**Figure 6:**
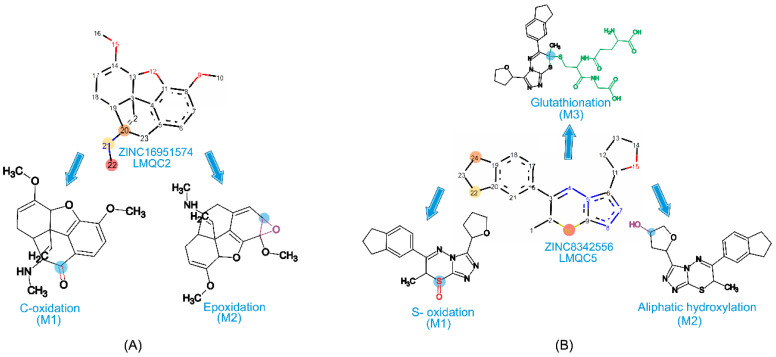
Prediction in Metatox with the metabolites and their respective chemical reactions of (**A**) ZINC16951574 (LMQC2) and (**B**) ZINC08342556 (LMQC5).

We used SMARTCyp [28] to evaluate the probable metabolites generated in phase I and II metabolisms undergone by the most promising compounds (ZINC16951574 (LMQC2) and ZINC8342556 (LMQC5)). The predictions for the compounds show that the following metabolic reactions occur: C-Oxidation, S-Oxidation, Aliphatic Hydroxylation, Epoxidation, and Glutathione, by the CYP 450 enzyme [29] (Figure 6 and Table 7).

The possible M1-1 metabolite of the compound ZINC16951574 (LMQC2) is formed by the C-oxidation reaction with an 82.83% probability of occurring. This metabolite had no side effects; however, it was carcinogenic in the liver in male mice and the lung in females.

The possible M2-1 metabolite of the compound ZINC16951574 (LMQC2) is formed by the Epoxidation reaction, with a 99.73% probability of occurring, to which the epoxide hydrolase catalyzes the trans addition of water that can develop during CYP450-dependent oxidation of aliphatic alkenes [30]. This metabolite presents arrhythmia and is carcinogenic in the urinary bladder in male rats and mice.

**Figure 7:**
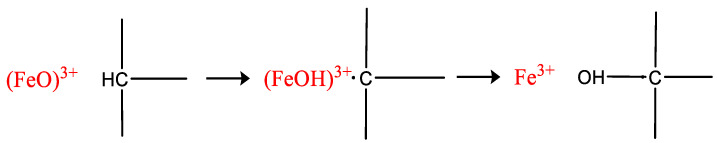
P450 enzymes preferentially catalyze hydroxylation.

The oxidation of some aliphatic alkenes produces metabolites that are sufficiently reactive to bind to the heme portion of cytochrome P450 covalently [29]. M1-2 metabolite showed an S-Oxidation of the compound ZINC8342556 (LMQC5), a reaction with a probability of occurring at 98.75%, and did not have any side effects. The M2-2 metabolite, with the chance of occurring at 99.65%, shows an aliphatic hydroxylation reaction that involves the insertion of oxygen in a C-H bond [29] (Figure 7). In the case of simple, straight-chain hydrocarbons, aliphatic hydroxylation occurs in the terminal methyl groups and the internal methylene groups.

The M3-2 metabolite, with a probability of occurring at 99.50, showed the Glutathionation reaction and a possible side effect of heart failure, with a probability of being carcinogenic in the stomach in female mice and male and carcinogenic in the vascular system in female rats.

For the prediction of the M3-2 metabolite, which is a Phase II reaction formed by the conjugation of xenobiotics with the tripeptide glutathione, which is composed of glycine, cysteine, and glutamic acid [30]. The conjugation of xenobiotics with glutathione fundamentally differs from its conjugation with other amino acids and dipeptides [29,31]. The M3-2 metabolite did not have any side effects, but it is likely carcinogenic in male mice’s kidneys and skin.

#### 2.7.2. ADME/Tox for Metabolism Prediction of the Most Promising Compounds

PreADMET was used to evaluate the ADMETox properties of metabolites generated from ZINC16951574 (LMQC2) and ZINC8342556 (LMQC5). All results obtained were compared to GNT (control) (Table 8).

Human intestinal absorption (HIA) and in vitro cell penetrability using the Caco2 cell model were used to describe intestinal drug absorption. All metabolites of the compounds ZINC16951574 (LMQC2) and ZINC8342556 (LMQC5) showed to be well absorbed (HIA ≥ 70%).

The results of PCaco-2 showed an average value of 22.9435 nm/s and hence can be classified as average permeability (value between 4 and 70 nm/s). As for renal clearance, all metabolites of ZINC16951574 (LMQC2) and ZINC8342556 (LMQC5) showed medium permeability (>4–70 nn/s). The compounds show negative Pskin values; they will not be administered transdermally. Yet they can be considered as topical actions, for example, repellents.

The distribution properties used were plasma protein binding (PPB) and brain/blood partition coefficient (BBB (C_Brain_/C_Blood_)) (see Table 9). The values of PPB ranged from 29.66% and 96.36%, where the metabolites showed weakly linked with plasma proteins with a value less than 90%, except for the metabolites M1-2 and M3-2 of compound ZINC8342556 (LMQC5) that were strongly bound to plasma proteins (PPB > 90%).

The penetration through the blood-brain barrier is a critical factor in the pharmaceutical field, and inactive compounds in the CNS (central nervous system) must not cross to avoid side effects [32]. The metabolites presented a CBrain/CBlood value of less than 1, characterizing inactive effects on the CNS. We used Metatox to evaluate the compounds’ toxic, carcinogenic, and mutagenic properties (Table 10).

The carcinogenicity test aims to identify the carcinogenic potential in animals and assess the risk in humans. The prediction in the rat for the metabolites of the compounds ZINC16951574 (LMQC2) and ZINC8342556 (LMQC5) showed evidence of carcinogenic activity. In mice, only the metabolites M1-1 and M1-2 of the compound LMQC2 obtained a positive prediction; there is no evidence of carcinogenic activity. The other metabolites of the LMQC5 compounds showed a negative prediction, which may characterize carcinogenic activity. When analyzing the mutagenicity, all the metabolites of the compounds LMQC2 and LMQC5 showed mutagenic predictions. Our results of metabolism predictions showed that the promissory molecules exhibited only one probability of the event happening, and that needs to be confirmed experimentally.

### 2.8. Molecular Docking and FTMap

#### 2.8.1. Binding Mode Interaction

The best compounds selected from the ADMETox and PASS analyses (ZINC16951574–LMQC2 and ZINC08342556–LMQC5) were investigated by docking to evaluate their binding mode with hAChE, an important molecular target against AD.

The validation of the molecular docking protocol was performed by comparing the position of the crystallographic inhibitor (GNT) (Table 11) and the position of fitting obtained for the same inhibitor.

According to the literature, a docking protocol must obtain an RMSD equal to or less than 2 angstroms between the crystallographic ligand and the redocked one [33,34,35]. The RMSD obtained between the crystallographic GNT and the one obtained by redocking was 0.07 Å (Figure S2). Moreover, redocking showed a binding affinity (ΔG) value of −9.90 kcal/mol. The theoretical binding affinity was considered close to the experimental value of −9.99 kcal/mol (Table 7 and Figure S3). Indicating that our docking protocol is satisfactory for evaluating the molecular binding mode of this type of complex.

**Table 11:** Comparison between experimental and theoretical binding affinities.

Receptor (CODE PDB ID)	Ligand	Experimental Binding Affinity * (kcal/mol)	Ki (nM) *h*AChE	Docking Predicted Binding Affinity (kcal/mol)	Resolution (Å)
4EY6	GNT	−9.99	61.96 × 10^−9^ [36]	−9.90	2.40

* Values calculated from experimentally determined inhibition constants (Ki), reported in the PDB and Peng et al. 2012 [37], according to Equation: ΔG = R × T × lnKi, where R (gas constant) is 1.987 × 10^−3^ kcal·mol^−1^·K^−1^ and T (temperature) is 303 K.

The molecular docking of ZINC16951574 (LMQC2) and ZINC08342556 (LMQC5) were evaluated based on the values of binding affinity, binding free energy, and visual inspection of the chemical structure, as well as the interactions with the amino acid residues using GNT as a control. ZINC16951574 (LMQC2) shows an elevated binding affinity value of −9.20 kcal/mol, followed by ZINC8342556 (LMQC5) with −10.00 kcal/mol, with a variation of ±0.7 and ±0.1 kcal/mol, respectively (Figure S3). The analysis of binding affinities calculated here indicated that ZINC16951574 (LMQC2) and ZINC8342556 (LMQC5) showed good results when compared to GNT, the most promising compound for potential inhibition of the enzyme AChE.

**Figure 8:**
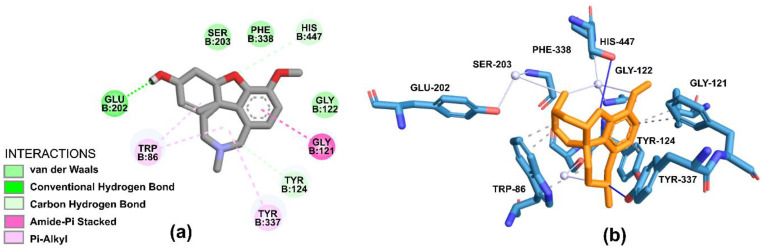
Representation of interactions in (**a**) 2D and (**b**) 3D obtainable through molecular docking for GNT. Light blue carbon atoms, dark blue nitrogen atoms and red oxygen atoms.

The GNT exhibits van der Waals and hydrophobic interactions. The van der Waals interactions with HIS-447B, stacking interaction π with GLY-121B, with interaction π–alkyl with TYR-337B and TRP-86. Residues such as TRP-86B, GLU-202B, and TYR-337B appear to be involved in the process of hydrophobic interactions (Figure 8).

Whereas, in the case of GNT, interactions are favored mainly by the stacking formed against GLU-202B in bonding hydrogen with the nitrogen atom of the main chain.

**Figure 9:**
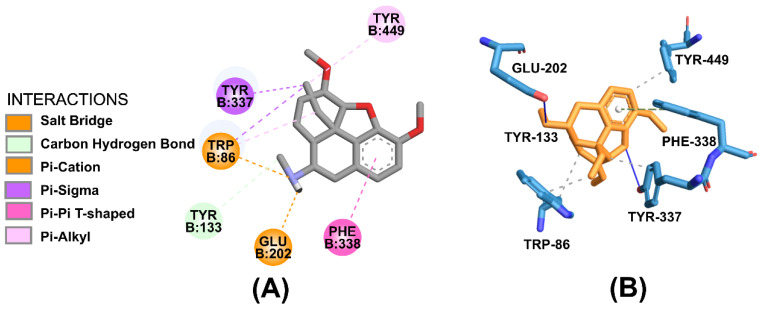
Representation of interactions in (**A**) 2D and (**B**) 3D obtainable through molecular docking for ZINC16951574 (LMQC2). Light blue carbon atoms, dark blue nitrogen atoms and red oxygen atoms.

ZINC16951574 (LMQC2) showed interactions of the saline bridge of the electrostatic binding type, where the NH_2_ group of the ligand interacts with oxygen from the amino acid GLU-202B (in addition to the interaction of π–Cation with GLU-202B and TRP-86B). The interaction between the OH of the amino acid TYR-337 and the carbon of the ligand is a Carbon-hydrogen bond where the carbon is the donor and the OH of the amino acid is the acceptor (Figure 9).

**Figure 10:**
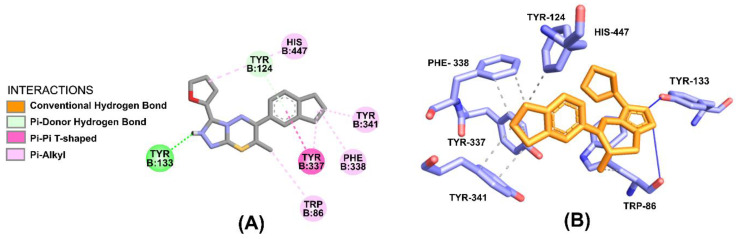
Representation of interactions in (**A**) 2D and (**B**) 3D obtainable through molecular docking for ZINC08342556 (LMQC5). Purple carbon atoms, dark blue nitrogen atoms and red oxygen atoms.

ZINC16951574 (LMQC2) showed stacking interactions π with TYR-133B and TYR-124B, while TYR-337B with interaction π–π and PHE-338B, TYR-341B, TRP-86B, and HIS-447B made hydrophobic interactions π–alkyl (Figure 10).

The conclusion made from these molecular docking followed by binding affinity and free binding energy calculations with a detailed analysis for the *h*AChE enzyme complex with the Ligand revealed a pattern of binding similar to that of GNT for our best-rated compounds obtained from virtual screening. These connect at the bottom of the active site, covering important binding and contacting peripheral anionic site residues [14].

In addition, a classic π-π stacking interaction was also observed in the ZINC16951574 (LMQC2) and ZINC08342556 (LMQC5), which act as an anchor to hold the compounds in their current positions beside a series of hydrogen bonds.

#### 2.8.2. Active Site Mapping—Fragment Binding Hot Spots

**Figure 11:**
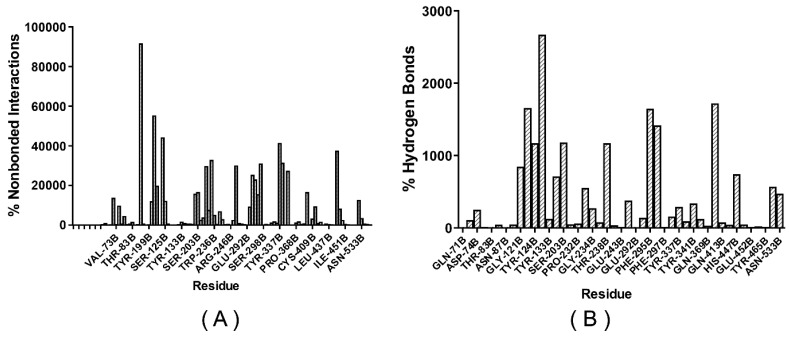
Connection at the site of binding: Distribution of unbound interactions between residues and probes in the consensus (CS) shown from the mapping of the crystalline structure *h*AChE (PDB ID: 4EY6) (**A**) Intermolecular interactions not linked between probes and waste; (**B**) Hydrogen bonds, exhibiting five important interactions GLY-121B, TYR-124, GLU-202, TYR-337B, and HIS-447B.

**Figure 12:**
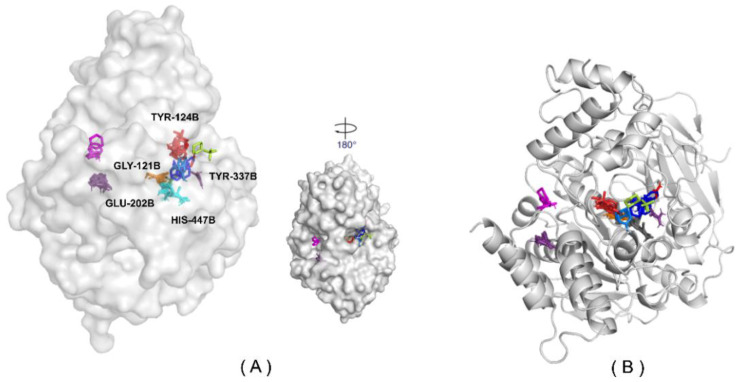
The FTMap identifies a cluster number per protein at the active site; the crystallographic structure of the *h*AChE protein is shown in the surface representation. (**A**) The FTMAP identified the hot spot region on the surface. (**B**) Two hot spots were identified and displayed on the ribbon line.

To compare our results with the literature data and those obtained by pharmacophore and molecular docking, we assess the main amino acid residues used as a robust and widely popular ‘hotspot’ detecting tool. The FTMap has successfully identified site B with the best-rated consensus (CS) for the *h*AChE. Moreover, it was found to have a very high probe cluster count, which indicates druggability [38,39]. Figure 11 and Figure 12 show the frequency of contacts between the probe molecules and each residue at the site of binding to the *h*AChE crystalline structure. As directed, GLY-121B, TYR-124B, GLU-202B, TYR-337B, and HIS-447B participate in the most significant number of interactions with the probe’s molecules. Several other probes also form hydrogen bonds. These are the same residues that ZINC16951574 (LMQC2) and ZINC08342556 (LMQC5) interacted with in our molecular docking results (Figure 8 and Figure 9). This suggests that the main compounds selected by this criterion may have great potential to act as inhibitors of the enzyme *h*AChE.

### 2.9. MD Simulations and Affinity Energy Calculations

#### 2.9.1. MD Simulations

The MD simulations were performed to study the binding strength and mode of action of the GNT, ZINC16951574 (LMQC2), and ZINC08342556 (LMQC5) interacting with *h*AChE, molecular target for DA. The trajectories analysis of MD assisted in evaluating the conformation changes with time in the protein-ligand complexes and control compounds. We have evaluated the dynamic change by means of RMSD, RMSF, and the radius of gyration (R_g_) during the 100 ns simulation period.

**Figure 13:**
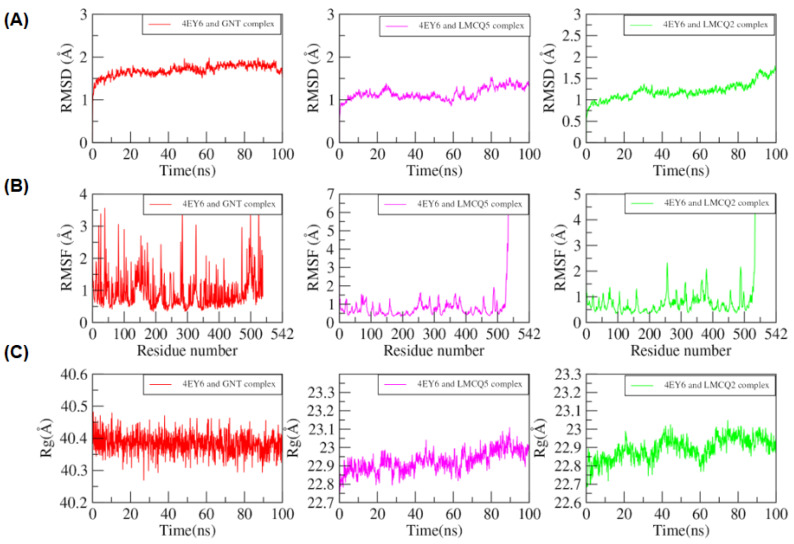
(**A**) Root-mean-square deviation RMSD plots of *h*AChE/GNT (control), *h*AChE/ZINC08342556 (LMQC5), and *h*AChE/ZINC16951574 (LMQC2) for 100 ns MD-simulation. (**B**) Root-mean-square fluctuation (RMSF) plots of *h*AChE/GNT (control)*, h*AChE/ZINC08342556 (LMQC5), and *h*AChE/ZINC16951574 (LMQC2). (**C**) the radius of gyration (Rg) plots of *h*AChE/GNT (control), *h*AChE *h*AChE/ZINC16951574 (LMQC2), and *h*AChE/ZINC08342556 (LMQC5). For all figures, the red color represents ZINC16951574 (LMQC2), and the green color represents ZINC08342556 (LMQC5).

Root-mean-square deviations (RMSD) were plotted by the mean of the carbon alpha (Cα) atom of the protein backbone. The RMSD value for the protein in the complex with ligands was approximately 3 Å as shown (Figure 13).

Figure 13A shows that the RMSD value starts steadily increase from 1 Å to 2 Å for the simulation time scale (0 to 10 ns) and then keeps fluctuating between 1.5 Å to 2 Å during the whole simulation time. The point fluctuation RMSD of both complexes is 1.49 ± 0.17 nm. No greater fluctuation was observed during whole trajectories, which showed that ligands remained bound to the protein’s active site and are stable to compounds ZINC16951574 (LMQC2), and ZINC08342556 (LMQC5) at 1.49 Å ± 0.17.

RMSF of all the residues was calculated to investigate the local protein mobility. As seen in Figure 13B, the protein’s ligand-binding site region is more rigid than the end terminal of the domain. Ligands binding residues (GLU202, TRP86, TYR337, Gly121, Gly122, and His447) are more rigid than other protein portions. A greater fluctuation is observed in Residue GLY256, CYS257 of the LMCQ5 complex since residue 256, 257 appears in the target protein’s loop region and does not impair the interaction of the ligands’ inactive site.

The radius of gyration (R_g_) values over the simulation time scale are calculated for complexes and are presented in Figure 13C. It has been observed that the R_g_ value ranges from 22.8 Å to 23.3 Å in a complex (*h*AChE/ZINC16951574-LMQC2) and 22.7 Å to 23.1 Å in a complex (*h*AChE-ZINC08342556/LMCQ5) compared with *h*AChE/GNT (control) and remain constant with little fluctuating between these ranges in the whole trajectory.

**Figure 14:**
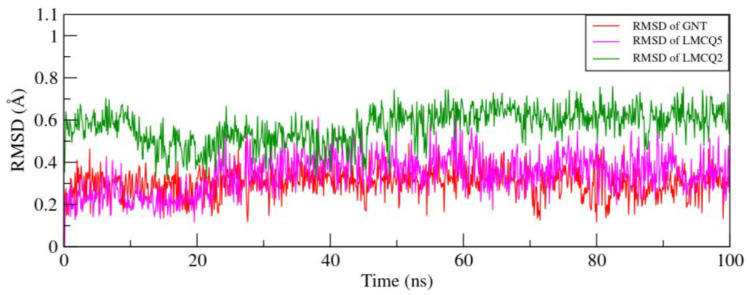
Root-mean-square deviation RMSDs plot of ligands GNT (control), ZINC08342556 (LMQC5), and ZINC16951574 (LMQC2) for 100 ns MD simulations.

Ligands generally showed a stable interaction profile throughout the MD simulations period. Moreover, the RMSD of only ligands was plotted to evaluate the interaction of ligands in the binding pocket (Figure 14). A peak of the RMSD of selected compounds and control is obtained between 0 Å and 1 Å, revealing that ligands (ZINC08342556-LMQC5, and ZINC16951574-LMQC2), showed a similar interaction pattern to observe for GNT (control compound) in the target protein.

#### 2.9.2. MMPBSA Binding Free Energy Calculation

MMPBSA results rank the compounds ZINC08342556 (LMQC5) < ZINC16951574 (LMQC2) < GNT (control). In control, compound electrostatics is a major energy contribution, whereas, in other compounds, ΔE_vdW_ is dominant over other energies term. Moreover, for ranking, binding energy criteria are used as a component of the energies ΔE_vdW_ and ΔEelectrostatic; the polar and non-polar contributions are ΔE_EPB_ and ΔE_NPOLAR_ (Table 12 and Figure S4).

ZINC16951574 (LMQC2) and ZINC08342556 (LMQC5) show stronger and more significant free energy values compared with GNT (Template). In comparison, ZINC08342556 (LMQC5) has the highest ΔE_vdW_, ΔEelectrostatic, and ΔE_EPB_ values. While ZINC16951574 (LMQC2) has the lowest ΔG_total_ due to the lowest electrostatic contribution, all compounds show favorable binding interaction and have significant binding energy values with the target protein. The ΔE_EPB_ also remained significantly unfavorable, showing that ΔE_vdW_ has a major contribution to binding with the target protein.

The total energy of the ZINC16951574 (LMQC2) and ZINC08342556 (LMQC5) is significantly different from that of control compounds GNT (control). In particular, the ZINC08342556 (LMQC5) complex shows a higher energy value of −54.2359 kcal/mol, indicating stronger interactions with receptor proteins in the active site. Other energy components include polar and non-polar solvation and van der Waals interaction forces. All ligands had stability during simulation time and showed negative binding energy values.

The energy components ΔE_vdW_ and ΔE_electrostatic_ were more involved in all complexes. In contrast, ΔE_EPB_ polar energy value 135.865 was observed in ZINC16951574 (LMQC2), which is significantly higher than the control compound (GNT), which showed that this compound has more polar interactions within the active site of the receptor. Additionally, ΔE_NPOLAR_ was found to be very similar in control and screening compounds. This indicates that ZINC16951574 (LMQC2) and ZINC08342556 (LMQC5) can be strong inhibitors of the *h*AChE enzyme receptor.

### 2.10. Prediction of Lipophilicity and Water Solubility and Structure-Activity Relationship of the Promising Molecule

Before proposing molecules’ theoretical synthetic routes for the compounds, we believe this methodological proposal will help medicinal chemists elaborate solutions based on their solubility, considering future in vivo tests [40].

Lipophilicity is a key parameter in drug discovery, based on the complements of the successful and informative physicochemical properties in medicinal chemistry [41]. Additionally, a drug meant for parenteral usage must be highly soluble in water to deliver a sufficient quantity of active ingredients in the small volume of such a pharmaceutical dosage [42].

Two topological methods for predicting water solubility are included in SwissADME. The first is an implementation of the ESOL36 model, and the second is an adaptation by Ali et al. 2012 [43]. The third SwissADME predictor for solubility was developed by SILICOS-IT [42].

The LogP parameter affects the ability of a molecule to decompose in non-polar versus aqueous environments [44]. GNT showed the highest consensual LogPo/w with a value of 3.33 (see Table 13), low solubility in water, and requiring organic solvents for solubilization. GNT has been reported to be soluble in organic solvents such as ethanol, DMSO, and dimethyl formamide (DMF) and is sparingly soluble in aqueous buffers [45,46,47].

The promising molecules investigated in the present study, the ZINC16951574 (LMQC2), presented (1.91) the lowest consensus value of LogPo/w. Then, ZINC08342556 (LMQC5) showed a value of 2.82 of LogPo/w. A range with the template molecule of ±1.42 and 0.51, respectively. Positive values indicate that all highly lipophilic molecules meet an essential criterion for a drug candidate.

To estimate water solubility, we can classify on a qualitative scale based on the log S scale: highly soluble > 0 > very soluble > −2 > soluble > −4 > moderately soluble > −6 > slightly soluble > −10 > insoluble [48]. Compounds ZINC16951574 (LMQC2) and ZINC08342556 (LMQC5) are moderately soluble (Ms) or soluble (S) in at least two solubility methods compared to GNT (control). In this study, only negative logS values in the range −6.00 to −6.75 were found to be promising compounds that showed consensus regarding logS values in the range, as shown in Table 14.

At the end of the virtual screening, the two compounds with the most promising results were subjected to a search on SciFinder^®^ (https://scifinder.cas.org/ (accessed on 30 September 2022)). No additional information on the compounds selected in the research was found; only information on some physical and chemical properties was already reported in the ZINCPharmer database. The ZINC16951574 (LMQC2) molecule has a characteristic dimethoxy group, very similar to a new class of medicines developed as a treatment for senile dementia of Alzheimer’s type. Its action increases central cholinergic activity, inhibiting AChE (AChE) in the brain [49].

The ZINC08342556 (LMQC5) molecule has in its structure a triazolothiadiazine group, which can serve as MTDLs (New multipurpose targeted ligands), as is evident in in vitro and in vivo studies from Sagar et al. 2018 [50] Additionally, it revealed important anti-amyloid, neuroprotective and anti-amnesic properties [50].

In general, the results of the present study suggest that the selected compounds can be tested for biological activities with good evidence of reproducing the results in silico. Therefore, future studies are needed to confirm the inhibitory activity of the AChE enzyme by these molecules.

### 2.11. Prediction of Synthetic Accessibility (SA) and Theoretical Synthetic Routes

#### 2.11.1. Prediction of Synthetic Accessibility

AMBIT and SwissADME were used to evaluate the SA of ZINC16951574 (LMQC2), and ZINC08342556 (LMQC5) (see Table 15).

Only the ZINC08342556 (LMQC5) molecule was the only one that presented the synthetic accessibility predicted as easy, getting a score of 56.318. ZINC16951574 (LMQC2) showed SA, achieving a score above 36.045, indicating median accessibility for synthesis. By comparison, the accessibility prediction for GNT (control) was 64.3331.

The result obtained by SwissADME for the ZINC16951574 (LMQC2) presented an SA score of 40.57%, and the ZINC08342556 (LMQC5) presented a score of 40.55% (Table 15). Compared to the template composite (GNT), which presented an SA score of 40.76%, the SA values were close, ranging between ±0.19 and ±0.21.

ZINC16951574 (LMQC2), and ZINC08342556 (LMQC5) can be considered difficult to synthesize, considering the results obtained and the data found in the literature [42].

**Figure 15:**
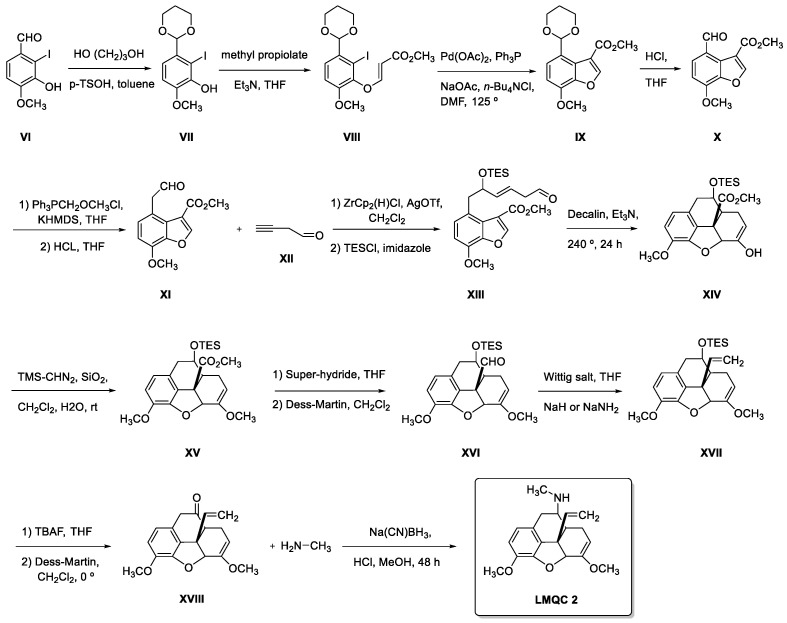
Theoretical synthetic route for the preparation of compound ZINC16951574 (LMQC2).

**Figure 16:**
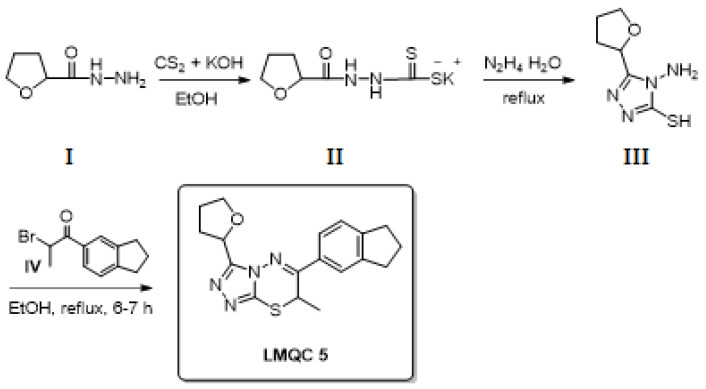
First theoretical synthetic route for the preparation of compound LQMC **5**.

With relation to GNT for the ZINC08342556 (LMQC5). Showed a variation of ±8.01 to AMBIT web server and ±0.21 to SwissADME, in which we propose in this study two theoretical synthetic routes; the first proposal is the formation of 4-amino-5-(tetrahydrofuran-2-yl)-4H-1,2,4-triazole-3-thiol (III) a key intermediate (Figure 15 and Figure 16). However, this step can provide the final compound in three stages with an excellent theoretical yield; we designed a second synthetic theoretical route composed of only a step to improve the yield of ZINC08342556 (LMQC5).

#### 2.11.2. Theoretical Synthetic Routes Proposed for Compounds ZINC16951574 (LMQC2), and ZINC08342556 (LMQC5)

The theoretical proposed synthesis of ZINC16951574 (LMQC2) (see Figure 15) starts with the formation of benzofuran acetaldehyde intermediate XI according to studies by G. Stork et al. 2009 [51]. Subsequently, protected dienyl XIII can be formed by a hydrozirconation of XII with Schwartz reagent, in situ reaction with XI, and finally trapping with chlorotrimethylsilane (TESCl).

Cycloaddition of XIII with decalin and triethylamine (Et_3_N) will produce phenanthrofuran XIV [51]. Conversion of the hydroxyl group in XIV to the methoxy group can be carried out by standard methylation to form XV [52]. Styrene XVII will be synthesized by reacting with Wittig salt under strongly basic conditions [53] from aldehyde XVI previously furnished from ester XV.

The deprotection of XVII and following Dess-Martin oxidation will allow obtaining the precursor XVIII. To conclude, reductive amination of carbonyl [54] XVIII will furnish the desired final product ZINC16951574 (LMQC2).

We propose two theoretical synthetic routes for the compounds ZINC08342556 (LMQC5). First (Figure 16), the formation of 4-amino-5-(tetrahydrofuran-2-yl)-4H-1,2,4-triazole-3-thiol (III) as a key intermediate is proposed. The readily available hydrazide I first reacted with carbon disulfide (CS2) in alcoholic potassium hydroxide (KOH) to give dithiocarbazate derivative II. Triazole III will then be formed from the reaction of II with hydrazide hydrate (N_2_H_4_ . H_2_O) under reflux conditions [55,56]. Materials I, IV, V, VI, and XII are commercially available. Finally, treating III with substituted phenacyl bromide IV under reflux and using ethanol (EtOH) as solvent [56] will produce the final triazolothiadiazine ZINC08342556 (LMQC5).

**Figure 17:**
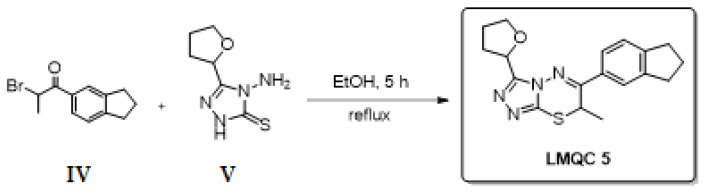
Second synthetic theoretical route (alternative) for the preparation of the compound LQMC5.

Although this proposed synthetic route can give the final compound in three steps with a good theoretical yield, we designed a second synthetic theoretical route consisting of only one step to improve the yield of ZINC08342556 (LMQC5) (see Figure 17). Cyclocondensation of V with substituted phenacyl bromide IV under reflux conditions in an ethanolic medium [57] will form the target compound ZINC08342556 (LMQC5). Materials I, IV, V, VI, and XII are commercially available. II

## 3. Materials and Methods

### 3.1. Compound Selection

Initially, GNT was selected as a template compound and 15 structures based on studies carried out by Atanasova et al., 2015 [14] (Table 16 and Figure S1) which has inhibitory activities (IC_50_) against the *h*AChE enzyme determined under the same biological test conditions, were divided into two classes according to their activities: (−) less active (those with IC_50_ ≥ 99.1 nM) and (+) most active (those with IC_50_ < 94.1 nM). The chemical structures of these selected inhibitors were designed using ChemSketch 12 and subsequently optimized using Discovery Studio 4.0.

### 3.2. Pharmacophore Model Generation

PharmaGist [58] generated the pharmacophoric pattern of GNT and 15 structures. The method essentially aligns and superimposes the molecules with the template (GNT), identifying the molecules with the greatest number of common characteristics and highest punctuation values, being considered the best candidates for pharmacophoric models [59]

### 3.3. Validation of the Pharmacophore Model

The pharmacophoric models indicated by PharmaGist were characterized according to their physical-chemical and structural properties, such as Atoms (ATM), Spatial characteristics (SF), Aromatic (ARO), Hydrophobic (HYD), and Hydrogen-bond acceptor (ACC). Pearson’s correlation was used to identify the relationship between the pharmacophoric properties of the 15 structures associated with the values of *p*IC_50_. The correlation cutoff point was 0.3, according to previous studies by Ferreira et al., 2019 [13] and Silva Costa et al., 2018 [60]. The experimental values of IC_50_ were converted into *p*IC_50_ (−log IC_50_) to reduce the inconsistencies caused by the statistical steps.

Hierarchical Cluster Analysis (HCA) was also used to verify and group the pharmacophoric models based on their chemical similarities. Euclidean distance was used to measure similarity. We used the most active structures (5.62 nM to 94.1 nM) and least active (99.1 nM to 222 nM) in the function of pIC50 (nM).

### 3.4. Pharmacophoric-Based Hierarchical Virtual Screening

The pharmacophoric model was submitted to ZINCpharmer [16] to discover new potential ligands, and the following databases were used: Zinc_Purchasable, Zinc_Drug Database, Zinc_In_Man, Zinc_Natural_Derivatives, Zinc_Natural_Products and Zinc_FDA_BindingD of the ZincPharmer database.

The screening process was carried out using the filters: (a) a number of different orientations of the same conformation (max hits per conf): 1; (b) a number of different orientations of different conformations of the same molecule (max hits per mol): 1; (c) molecular weight: 200 to 500 DA; (d) top hit 200; and (d) number of rotatable bonds in the molecule: 1 to 10.

### 3.5. In Silico Evaluation of Pharmacokinetic and Toxicological Properties of Promising Compounds

ADMETox and hepatotoxicity properties were evaluated using software such as QikProp, PreADMET, Derek, and ProTox-II. The pharmacokinetic properties were analyzed with QikProp: #star, Linpiski rule, human intestinal oral absorption; QPPCaco; QPPMDCK; QPlogPo/w; CNS; QPlogBB and MW. PreADMET calculates pharmacokinetic properties such as Human Intestinal Absorption (HIA), the permeability of CaCO_2_ cells in vitro (P_CaCO2_), the permeability of Maden Darby Canine Kidney cell (P_MDCK_), skin permeability (P_Skin_), plasma protein binding (PPB) and penetration through the blood/brain barrier (C_Brain_/C_Blood_), and toxicological properties such as mutagenic and carcinogenic effects. The toxicological properties were predicted using the Derek software 10.0. This software is based on the search for the 2D similarity of the molecule in question or its fragments with fragments from a database with already recognized toxicity (toxicophore). It correlates the structures with various toxicological characteristics, such as mutagenicity, carcinogenicity, skin sensitization, irritation, reproductive and developmental toxicity, neurotoxicity, etc. The compounds had their hepatotoxicity evaluated using ProTox-II [22]. It allows the enabling the prediction of the largest number of toxicity endpoints consisting of 33 models and their associated LD_50_ values.

### 3.6. Prediction of Biological Activity

The prediction of the biological activity of the compounds was performed using the PASS software [23], which predicts with a high accuracy of up to 2000 biological activities that are possible for chemical compounds. The approach used in the PASS is based on the suggestion that the chemical structure of compounds is closely related to their biological activity. The software was used to predict the potential biological activity of *h*AChE ligands against Alzheimer’s and cytochromes 3A4 and 2D6.

### 3.7. Molecular Docking Simulations and FTMap

#### 3.7.1. Docking Simulations

The crystallographic structure of hAChE interacting with GNT was retrieved from the Protein Data Bank (PDB), where it can be located with the PDB ID: 4EY6 [61] (see Table 17).

Molecular docking calculations were performed using AutoDock 4.2/PyRx [62,63] with parameters of the genetic algorithm (with a population size of 150), a maximum number of evaluations of 250,000, a maximum number of generations of 27,000, and a crossing rate of 0.8. Interactions between inhibitors and *h*AChE were visualized using Discovery Studio 4.0.

The calculation of the binding affinity (ΔG) was also carried out to compare the actual data collected and the values obtained in silico, which was the same protocol adopted by Santos et al., 2020 [14], according to Equation (1).

ΔG = −*RT* ln K_i_ (1)

where R (gas constant) is 1.987 × 10^−3^ kcal·mol^−1^·K^−1^, T (temperature) is 303 K, and Ki (inhibition constant) is 61.96 × 10^−9^ M for GNT (reported in the PDB) [61].

The best free energy of binding values was obtained in the PyRx tool GUI and log files. The calculation of free energy of binding that we will try to estimate is calculated according to Morris et al., 2009 [64] and Lai et al., 2012 [65] (Equation (2)).

ΔG_bind_ = ΔH − TΔS (2)

ΔH represents the enthalpic contribution, TΔS represents the entropic contribution, and T is the temperature in Kelvin. ΔS are connected via the Gibbs equation.

#### 3.7.2. Active Site Mapping—Fragment Binding Hot Spots

The FTMap [66] identifies protein druggable hot spots using Fourier domain correlation techniques; the FTMAP method consists of accelerated MD simulations to calculate and equilibrate the structure on which the surface is interacting with a series of probes. For the FTMap analysis, the crystalline structure *h*AChE in the complex with GNT was submitted and analyzed in the protein interaction mode, according to the published protocol [66,67].

### 3.8. Metabolites and Their Properties ADMETox and Carcinogenicity

The compounds ZINC16951574 (LMQC2) and ZINC08342556 (LMQC5) were investigated for the generation of metabolites; ADMETox and carcinogenic properties of these metabolites using SMARTCyp [28], PreADMETox [68], and Metatox [69].

### 3.9. MD Simulations

The MD simulations were run using MDWeb, based on simulation software such as NAMD, Amber, and Gromacs. The complexes that were submitted to MD simulations were obtained via the molecular docking of the ligands GNT, LMCQ5, and LMCQ2 with *h*AChE as molecular targets. The parameters of the ligands were defined by GAFF [70], proteins were then treated with FF14SB [71], and the ionization state of their amino acids was studied with PROPKA [72] at a neutral pH. The complexes were solvated in an octahedral periodic box dimension of 12 Å defined by the TIP3P [73] water model. The partial charges of the complexes were neutralized by adding counter ions.

The energy of the systems was mimicked in five stages, where each one was executed 3000 cycles using the steepest descent method and 5000 cycles using the conjugate gradient algorithm. In the first stage, the hydrogen of the water molecules was minimized, and in the second stage, the water molecules and ions were minimized soon after the hydrogen atoms of the protein were minimized, and finally, all the solvents and solutes had their energy minimized.

The systems were heated gradually to 300 K for 800 ps and were performed using an NVT ensemble. The collision frequency used was 3 ps^−1,^ and the Langevin thermostat was used for temperature control [74]. The particle mesh Ewald method [75] was used to calculate electrostatic interactions, and the bonds involving hydrogen atoms were restricted by means of the SHAKE algorithm [76].

### 3.10. Binding Free Energy Calculation

The affinity energy of *h*AChE interacting with GNT, LMCQ5, and LMCQ2 was calculated using Molecular Mechanics/Poisson–Boltzmann Surface Area (MM/PBSA) [77,78,79].

The free energy was estimated according to the following (Equation (3)):

ΔG_bind_ = ΔE_MM_ + ΔGs_olv_ − TΔS (3)

ΔG_bind_ is the affinity energy resulting from the sum of the total energy in the gas phase (ΔE_MM_), the free energy of solvation (ΔG_solv_), and entropy (TΔS).

ΔE_MM_ is the sum of ΔE_internal_ (connections, angles, and dihedra), ΔE_electrostatic_ (electrostatic contributions), and ΔE_vdW_ (van der Waals contributions), according to the following (Equation (4)):

ΔE_MM_ = ΔE_internal_ + ΔE_electrostatic_ + ΔE_vdW_ (4)

ΔG_solv_ can be obtained by solving (Equation (5)):

ΔG_solv_ = ΔG_PB_ + ΔG_SASA._ (5)

when the polar contributions (ΔG_PB_) are calculated using either the PB model or the non-polar contributions (ΔG_SASA_), they were determined from the calculation of the solvent accessible surface area (SASA).

### 3.11. Prediction of Lipophilicity and Water Solubility

The evaluation of the lipophilicity and water solubility of the ZINC16951574 (LMQC2) and ZINC08342556 (LMQC5) compounds was performed using the SwissADME based on the methodological proposal by Ramos et al., 2022 [44] and dos Santos et al., 2022 [80].

### 3.12. Theoretical Prediction of Synthetic Accessibility (SA) for Promising Compounds

The prediction of SA of ZINC16951574 (LMQC2) and ZINC08342556 (LMQC5) was performed using the AMBIT and SwissADME. AMBIT utilizes the model for SA and uses four weighted molecular descriptors, which represent different structural and topological features combined in an additive scheme. The algorithm calculates molecular complexity, stereochemical complexity, and complexity due to fused and bridged systems for a given target molecule or set of molecules. The SA is issued as a score ranging from 0 to 100, where 100 is the maximum synthetic accessibility; that is, the molecule is more easily synthesized [81]. SwissADME will be used for (SA), performing fragment-based forms of SA prediction. This value is a score based on the fragmented analysis of structures from more than 13 million compounds with the hypothesis that the more frequent a molecular fragment, the easier it is to obtain the molecule. The SA Score range is set between 10 (easy synthesis) and 100 (very difficult synthesis) [40].

## 4. Conclusions

In this paper, our study of the hierarchical clustering analysis (HCA) was adequate for validating the pharmacophoric model since it is possible to visualize the arrangement of the molecules about their structural similarities. FTMap identified the main hot spot of the *h*AChE crystalline structure with the best consensus location of the nine different ranks. This hot spot coincides with the *h*AChE B site, an essential site for GNT binding, confirming the binding site with the main amino acids from the results of promising compounds. MD simulations also confirmed the stable binding of ZINC16951574 (LMQC2), and ZINC08342556 (LMQC5) to *h*AChE. The MD simulations’ results strongly indicate that ZINC16951574 (LMQC2) and ZINC08342556 (LMQC5) can be strong inhibitors of hAChE enzyme receptors; ZINC08342556 (LMQC5) has the highest ΔE_vdW_, ΔE_electrostatic_, and ΔE_EPB_ values.

They were followed by metabolic predictions at the cytochrome site P450 and were less toxic with better ADME. The prediction of acute oral toxicity of GNT was comparable with experimental data. However, the acute toxicity of the presentations will require experimental validation of their metabolic products. In addition, the synthesis of these substances and design derivatives allows the pharmacophore to be established and the biological profile to be modulated, representing an excellent opportunity for the performance of synthetic and medicinal organic chemicals in the symptomatology of various diseases, including Alzheimer’s disease.

The analysis of water solubility and lipophilicity, as well elaboration Theoretical Synthetic Route of the promising compounds, will be essential for future tests for the inhibitory activity of *h*AChE to validate computational methods. Future studies can explore their biological activity. The ZINC16951574 (LMQC2) and ZINC08342556 (LMQC5) molecules showed a potential profile, suggesting satisfactory inhibitory properties for *h*AChE. Therefore, in silico drug retargeting can identify promising results that may be therapeutically useful in AD.

## Figures and Tables

**Table 1:** Result of the matrix with 6 descriptors and the Pearson correlation of the variables resulting from the virtual screening ligand-based molecules.

Structures	ATM	SF	ARO	HYD	ACC	*p*IC_50_
1	42	11	1	6	4	8.250
2	85	16	2	9	5	8.060
3	84	17	2	10	5	7.909
4	85	16	2	9	5	7.829
5	81	15	2	8	5	7.750
6	83	16	2	8	6	7.619
7	80	16	2	8	6	7.460
8	98	21	2	13	6	7.260
9	82	16	2	8	6	7.239
10	109	24	2	17	5	7.219
11	103	22	2	15	5	7.050
12	101	22	2	14	6	7.030
13	87	17	2	8	7	7.010
14	84	16	2	7	7	6.949
15	90	18	2	9	7	6.920
16	93	19	2	10	7	6.659
SF	0.928	-	-	-	-	-
ARO	0.806	0.541	-	-	-	-
HYD	0.802	0.944	0.335	-	-	-
ACC	0.338	0.187	0.501	−0.143	-	-
*p*IC_50_	−0.634	−0.607	−0.494	−0.355	−0.804	-

**Table 2:** Pharmacophore characteristics.

Pharmacophoric Characteristics		Coordinates			
		X	Y	Z	Radius
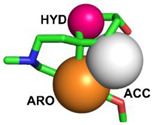	Aromatic (ARO)	8.20	−59.95	−3.93	1.1
	Hydrophobic (HYD)	8.49	−62.48	−25.88	1.0
	Hydrogen bond acceptor (ACC)	7.59	−61.43	−22.89	0.5

**Table 3:** Pharmacokinetic predictions for GAL and molecules obtained by virtual screening.

Compounds	#Star ^a^	RO5 ^b^	%HOA ^c^	QPP Caco ^d^	QPP MDCK ^e^	QPlog Po/w ^f^	CNS ^g^	QPlogBB ^h^	MW ^i^
		0–4	<25 Poor >80 Great	<25 Poor >500 Great	<25 Poor >500 Great	<5	−2 (Inactive) +2 (Active)	<1	150–500
GNT (control)	1	0	90.0	716.5000	381.7100	2.042	1	0.368	287.350
ZINC86196920	0	0	100	4677.623	2621.609	2.786	1	0.105	208.257
ZINC16951574 (LMQC2)	0	0	100	1838.444	1056.986	3.174	2	0.611	313.397
ZINC91960073	0	0	100	5925.536	7466.586	2.815	1	0.410	253.685
ZINC08342556 (LMQC5)	0	0	100	2928.309	2764.505	3.828	1	0.144	340.452
ZINC86199797	0	0	100	4673.581	4673.581	3.166	1	0.029	222.284
ZINC13108311	0	0	100	2387.068	5990.536	3.254	1	0.081	350.234
ZINC13362890	0	0	100	3705.456	2037.983	2.415	1	0.111	230.267
ZINC21657754	0	0	100	3792.205	2089.602	2.136	1	0.065	246.266

^a^ Number of computed properties that fall outside the required range for 95% of the known drug; ^b^ Number of violations of Lipinski’s ‘Rule of Five.’ ^c^ Percentage of human oral absorption (%HOA) (acceptable range: <25% is poor and >80% is high); ^d^ Predicted Caco-2 cell permeability in nm/s (acceptable range: <25 is poor and >500 is great); ^e^ Predicted apparent MDCK cell permeability in nm/s (acceptable range: <25 is poor and >500 is great); ^f^ the apparent permeability of compound between octanol/water (QPlogPo/w) < 5; ^g^ CNS Predicted activity of the central nervous system on a scale of −2 (inactive) to +2 (active). ^h^ the apparent permeability of compound in blood–the brain barrier (QPlogBB). ^i^ Molar weight.

**Table 4:** Toxicity prediction by toxicophoric identification of acute oral and hepatotoxicity.

Compounds	Toxicity Prediction Alert (Lhasa Prediction)	Toxicophoric Group		LD_50_ Predicted in Rodents (mg/kg)	Toxicity Class *	Hepatotoxicity
GNT (control)	-	-		85	III	Inactive
ZINC86196920	-	-		362	IV	Inactive
ZINC16951574 (LMQC2)	-	-		520	IV	Inactive
ZINC08342556 (LMQC5)	-	-		1000	IV	Inactive
ZINC13362890	-	-		360	IV	Inactive
ZINC86199797	-	-		362	IV	Inactive
ZINC21657754	-	-		800	IV	Active
ZINC13108311	Skin sensitization	Thiol or thiol exchange agent	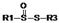	1000	IV	Active
ZINC15910273		Hydrazine or precursor	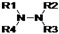	1000	IV	Active

* If swallowed = Class I: fatal (LD_50_ ≤ 5). Class II: fatal (5 < LD_50_ ≤ 50). Class III: toxic (50 < LD_50_ ≤ 300). Class IV: harmful (300 < LD_50_ ≤ 2000). Class V: may be harmful (2000 < LD_50_ ≤ 5000), Class VI: non-toxic (LD_50_ > 5000).

**Table 5:** Prediction of biological activity of compounds used for virtual screening using the PASS online server.

Compound	Pa	Pi	Biological Activity
GNT (control)	0.957	0.003	Substrate CYP2D6
	0.553	0.052	Substrate CYP3A4
	0.428	0.055	Treatment of Alzheimer’s disease
ZINC86196920	0.408	0.056	Substrate CYP2D6
	0.413	0.085	Substrate CYP3A4
	0.223	0.124	Treatment of Alzheimer’s disease
ZINC16951574 (LMQC2)	0.553	0.024	Substrate CYP2D6
	0.264	0.187	Substrate CYP3A4
ZINC08342556 (LMQC5)	-	-	-
ZINC86199797	0.544	0.025	Substrate CYP2D6
	0.532	0.055	Substrate CYP3A4
ZINC13362890	0.191	0.165	Treatment of Alzheimer’s disease
ZINC21657754	0.192	0.164	Treatment of Alzheimer’s disease

Pa = probability of being active (Pa > 0.000 or Pa = 1.000); Pi = Probability of being inactive (Pi = 0.000).

**Table 6:** Two-dimensional structures of the selected molecules after prediction of biological activity.

Compound	Structure	Smile
ZINC86196920	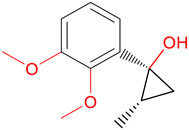	COc1cccc([C@]2(O)C[C@@H]2C)c1OC
ZINC16951574 (LMQC2)	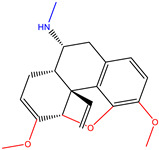	C=C[C@@]12c3c4ccc(OC)c3O[C@@H]1C(OC)=CC[C@@H]2[C@H](NC)C4
ZINC08342556 (LMQC5)	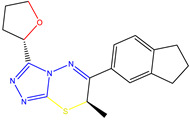	C[C@H]1Sc2nnc([C@@H]3CCCO3)n2N=C1c1ccc2c(c1)CCC2
ZINC86199797	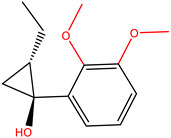	CC[C@H]1C[C@@]1(O)c1cccc(OC)c1OC
ZINC13362890	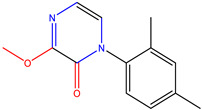	COc1nccn(-c2ccc(C)cc2C)c1=O
ZINC21657754	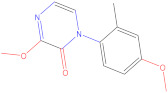	COc1ccc(-n2ccnc(OC)c2=O)c(C)c1

**Table 7:** Probable metabolites for ZINC16951574 (LMQC2) and ZINC8342556 (LMQC5), chemical reactions, and values of the probability of the reaction to happen.

ZINC16951574 (LMQC2)			
Metabolites	Phase Type	Chemical Reaction	Probability (%)
M1-1	Phase I reaction (Oxidation)	C-oxidation	82.83
M2-1		Epoxidation	99.75
ZINC8342556 (LMQC5)			
M1-2	Phase I reaction (Oxidation)	S-oxidation	98.75
M2-2		Aliphatic hydroxylation	99.65
M3-2	Phase II biotransformation reaction	Glutathionation	99.50

**Table 8:** Absorption properties of the metabolites of the most promising compounds.

Absorption and Excretion					
Compound	Metabolites	HIA (%)	PCaco-2 (nm/sec)	P_skin_ (cm/h)	P_MDCK_ (nm/sec)
GNT	-	95.402480	20.9301	−4.17647	78.0917
ZINC16951574 (LMQC2)	M1-1	95.758170	51.9435	−3.92615	283.166
	M2-1	95.874961	53.1166	−4.44136	57.8788
ZINC8342556 (LMQC5)	M1-2	99.307339	20.3666	−4.1935	80.2919
	M2-2	97.599836	23.6799	−4.41052	84.5731
	M3-2	95.758170	22.9435	−4.31616	84.0711

**Table 9:** Distribution of the properties of PPB and penetration of the blood-brain barrier to the metabolites of the most promising compounds.

Distribution			
Compound	Metabolites	PPB (%)	BBB (C_Brain_/C_Blood_)
GNT (control)	-	25.772647	0.578707
ZINC16951574 (LMQC2)	M1-1	44.430986	0.663151
	M1-2	29.666364	0.476563
ZINC8342556 (LMQC5)	M2-1	96.361373	0.96609
	M2-2	89.331353	0.246839
	M3-3	90.730886	0.233788

**Table 10:** Toxicological, carcinogenic, and mutagenic properties for the metabolites of the most promising compounds.

Compound	Metabolites	Carcinogenicity		Ames Test
		Mouse	Rat	Mutagenicity
GNT (control)	-	Negative	Negative	Mutagen
ZINC16951574 (LMQC2)	M1-1	Negative	Positive	Mutagen
	M1-2	Negative	Positive	Mutagen
ZINC8342556 (LMQC5)	M2-1	Negative	Negative	Mutagen
	M2-2	Negative	Negative	Mutagen
	M2-3	Negative	Negative	Mutagen

**Table 12:** Binding free energy (kcal/mol) of the compounds studied.

Compound	ΔG_total_ (a)	ΔE_VDW_ (b)	ΔE_electrostatic_ (c)	ΔE_EPB_ (d)	ΔE_ENPOLAR_ (e)
GNT (control)	−44.4665	−48.1589	−7.5486	15.7695	−4.5285
ZINC16951574 (LMQC2)	−52.3996	−46.3026	−135.8650	135.2977	−5.5297
ZINC08342556 (LMQC5)	−54.2359	−58.9034	−1.4877	11.1959	−5.0406

(a) ΔG_total_ (total free energy); (b) ΔE_VDW_ (van der Waals total free energy); (c) ΔE_electrostatic_ (electrostatics interactions); (d) ΔE_EPB_ (polar solvation free energy); (e) ΔE_NPOLAR_ (non-polar solvation free energy). GNT. Data are expressed as kcal/mol.

**Table 13:** Lipophilicity and Water Solubility for Promising Compounds *.

Compound	iLOG	XLOGP	WLOGP	MLOGP	SILICOS-IT	Consensus LogP
GNT (control)	3.37	3.36	2.66	3.38	3.87	3.33
ZINC16951574 (LMQC2)	2.64	1.84	1.32	1.74	2.03	1.91
ZINC08342556 (LMQC5)	3.27	2.85	2.57	2.13	3.25	2.82

* iLOGP: physics-based method relying on free energies of solvation in *n*-octanol and water calculated by the Generalized-Born and solvent-accessible surface area model; XLOGP: an atomistic method including corrective factors and knowledge-based library; WLOGP, implementation of a purely atomistic method based on the fragmental system of Wildman and Crippen; MLOGP: an archetype of a topological method relying on a linear relationship with 13 molecular descriptors; SILICOS-IT: a hybrid method is relying on 27 fragments and 7 topological descriptors.

**Table 14:** Prediction of solubility through the free web tool SwissADME *.

Compound	ESOL	Ali	SILICOS-IT	Consensus LogS
GNT (control)	−4.27 (ms)	−4.67 (ms)	−4.82 (ms)	−6.00 (ps)
ZINC16951574 (LMQC2)	−3.51 (s)	−3.34 (s)	−4.52 (ms)	−6.75 (ps)
ZINC08342556 (LMQC5)	−2.93 (s)	−2.34 (s)	−2.96 (s)	−6.19 (ps)

* Solubility (s = Soluble, ms = Moderately soluble, ps = Poorly soluble), and Consensus LogS.

**Table 15:** Synthetic Accessibility (SA) Prediction for Selected Compounds *.

Compound	SA (%) (a)	SA Score (%) (b)
GNT (control)	64.33	40.76
ZINC16951574 (LMQC2)	36.05	40.57
ZINC08342556 (LMQC5)	56.32	40.55

* Synthetic Accessibility (SA). (a) AMBIT web server ranges from easy accessibility (score ≥ 50), median accessibility (10 < score ≤ 49), and difficult accessibility (score ≤ 10). (b) SwissADME—SA scores range from 10 (very easy) to 100 (very difficult).

**Table 16:** Structures and IC_50_ values for the test set of GNT derivatives.

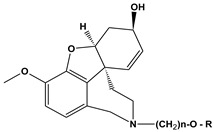 **(a)**							
**No**	**R (b)**	**n (c)**	**IC_50_ (d), nM**	**No**	**R (b)**	**N (c)**	**IC_50_ (d), nM**
**1**	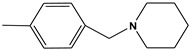	6	5.62	**9**	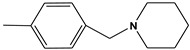	10	61.20
**2**	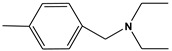	6	6.52	**10**	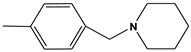	9	88.10
**3**	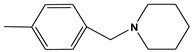	4	8.86	**11**	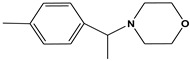	4	94.10
**4**	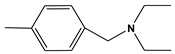	4	12.40	**12**	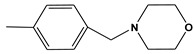	4	99.10
**5**	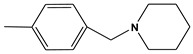	2	24.50	**13**	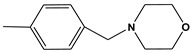	6	113.00
**6**	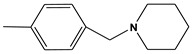	8	34.70	**14**	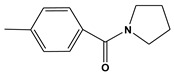	6	122.00
**7**	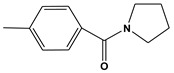	4	53.80	**15**	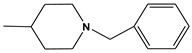	6	222.00
**8**	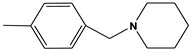	12	58.10	-	-	-	-

(a) The structures of the compounds, (b) Derivatives with indole moiety ending the side chain attached to N-atom, (c) Substituent fixed as an indole moiety ending group in combination with various C substituents, (d) Inhibitory concentration-50%.

**Table 17:** Protocol data used in the validation of molecular docking.

Receptor	Ligand	Coordinates of the Grid Center (Angstrom)	Grid Dimensions (Angstrom)
hAChE (PDB ID: 4EY6)	GNT	X = 8.817 Y = −60.624 Z = −23.964	X = 18.522 Y = 23.934 Z = 11.629

## Data Availability

Data available in this study are available in this article or on request from the corresponding author.

## Supplementary Materials

The following supporting information can be downloaded at: https://www.mdpi.com/article/10.3390/molecules28031035/s1, Figure S1: Structures and IC_50_ (nM) values for the test set of GAL derivatives. Figure S2: RMSD representation of the crystallographic ligand (green) and better molecular fitting position (red). Figure S3. The binding affinity of GNT and compounds resulting from the virtual screening of the *h*AChE enzyme receptor on the Protein Data Bank (PDB) ID 4EY6. Figure S4. Binding free energy of GNT and screened compounds complex with *h*AChE enzyme receptor on the Protein Data Bank (PDB) ID 4EY6.

## Sample Availability

Samples of the compounds are available from the authors.
