# Supplementary material for: Galantamine Based Novel Acetylcholinesterase Enzyme Inhibitors: A Molecular Modeling Design Approach
Source: Molecules. 2023 Jan 19;28(3):1035. doi: 10.3390/molecules28031035 (PMC9919016; doi:10.3390/molecules28031035)
Supplement: Supplementary file 1 [file molecules-28-01035-s001.zip › molecules-1975503-supplementary.pdf]

molecules

MDPI

*Supplementary Materials*

# Galantamine Based Novel Acetylcholinesterase Enzyme Inhibitors: A Molecular Modeling Design Approach

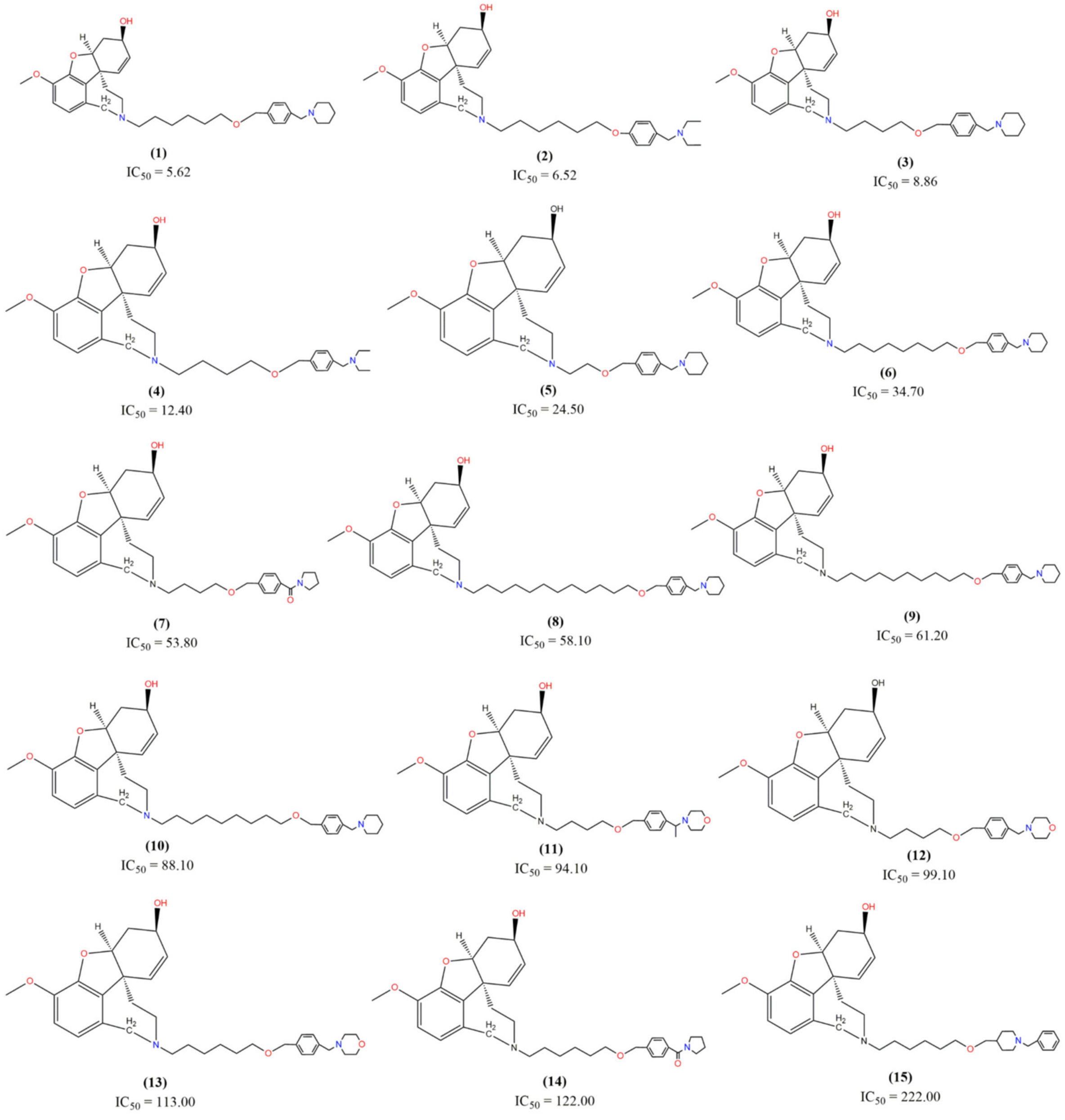


**Figure S1.** Structures and $IC_{50}$ values for the test set of GAL derivatives.

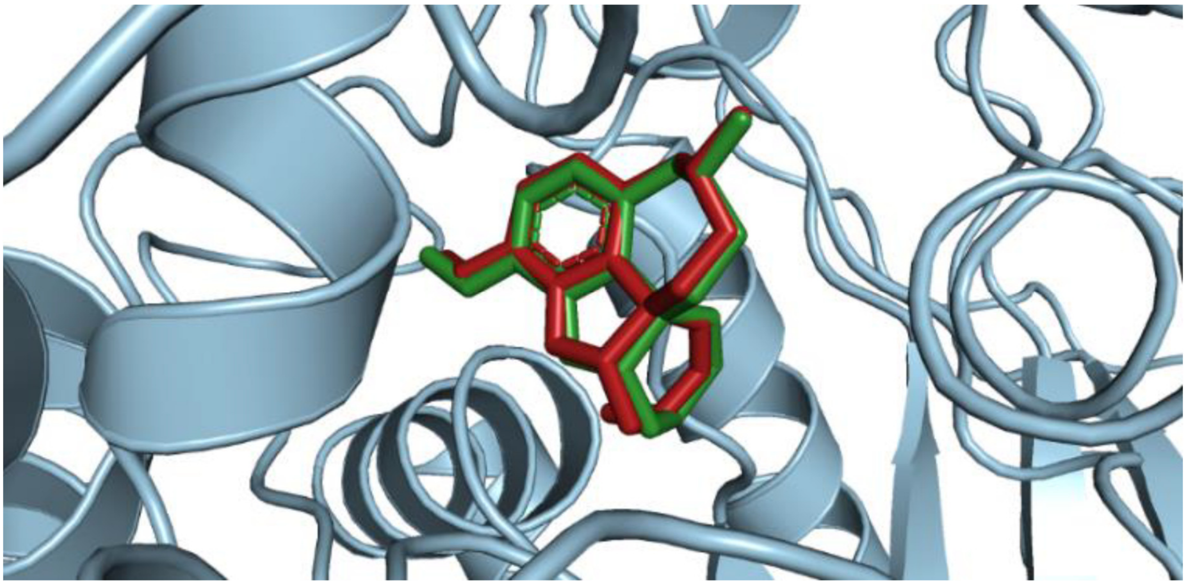

**Figure S2.** RMSD representation of the crystallographic ligand (green) and better molecular fitting position (red).

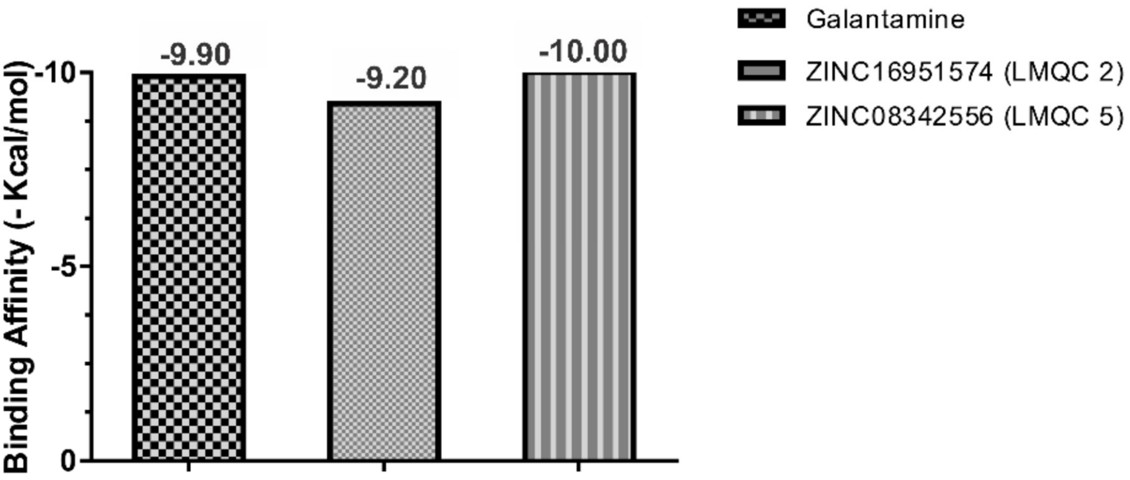


**Figure S3.** Binding affinity of Galantamine and compounds resulting from the virtual screening of the human acetylcholinesterase enzyme receptor on the Protein Data Bank (PDB) ID 4EY6.

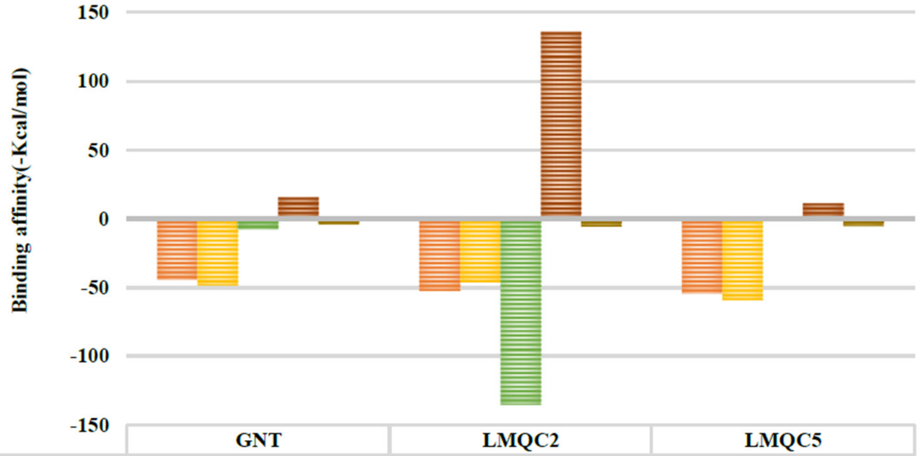


| | GNT | LMQC2 | LMQC5 |
|---|---|---|---|
| Free energy value (Kcal/mol) | -44.4665 | -52.3996 | -54.2359 |
| VDW | -48.1589 | -46.302 | -58.9034 |
| Electrostatics | -7.5486 | -135.2977 | -1.4877 |
| EPB | 15.7695 | 135.865 | 11.1959 |
| ENPOLAR | -4.5285 | -5.5297 | -5.0406 |

**Figure S4.** Binding free energy of GNT and screened compounds complex with human acetylcholinesterase enzyme receptor on the Protein Data Bank (PDB) ID 4EY6.
